# Supplementary material for: Active Lifestyle and Mobility of Adults with Vision Impairment: A Multiphase Mixed-Methods Study
Source: Int J Environ Res Public Health. 2023 Sep 27;20(19):6839. doi: 10.3390/ijerph20196839 (PMC10572964; doi:10.3390/ijerph20196839)
Supplement: Supplementary file 1 [file ijerph-20-06839-s001.zip › Supplemental Figure S1.pdf]

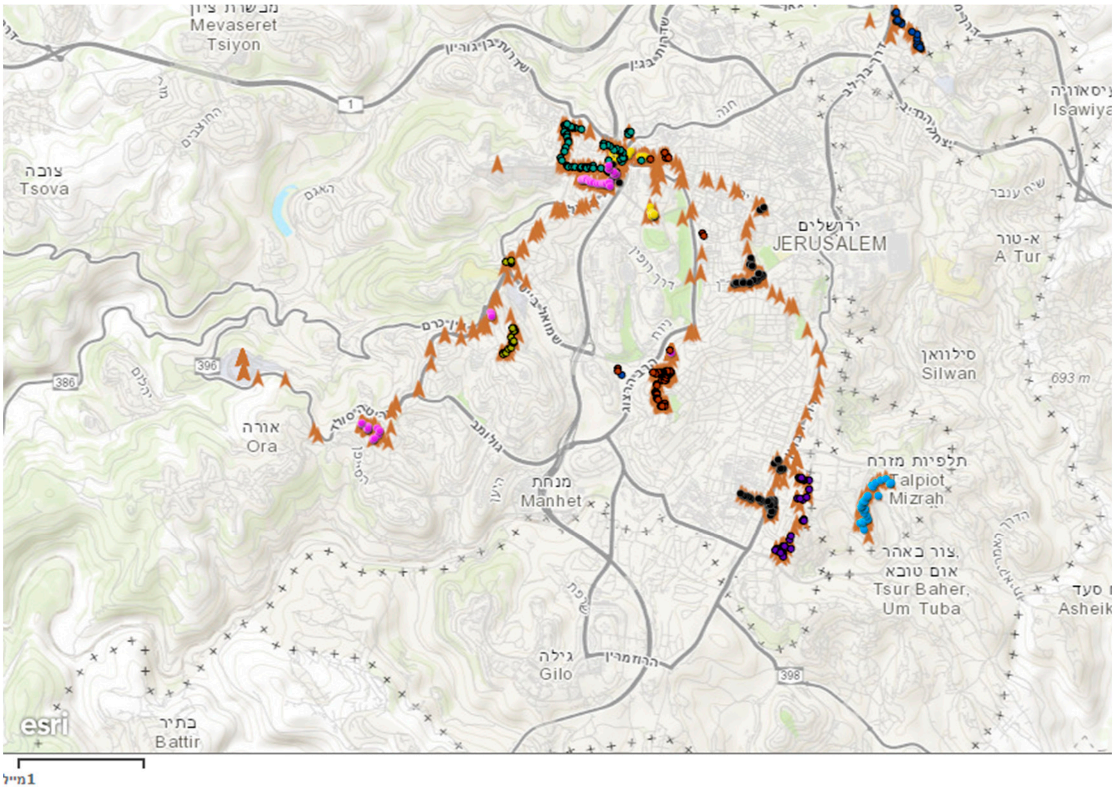

GPS tracking ● documented entities

Supplemental Figure S1. Tracking map of routes and documented entities
