# Supplementary material for: Active Lifestyle and Mobility of Adults with Vision Impairment: A Multiphase Mixed-Methods Study
Source: Int J Environ Res Public Health. 2023 Sep 27;20(19):6839. doi: 10.3390/ijerph20196839 (PMC10572964; doi:10.3390/ijerph20196839)
Supplement: Supplementary file 1 [file ijerph-20-06839-s001.zip › Supplemental Table S1.pdf]

Supplemental Table S1. Description of the documented Entities

Documented Entities	Systematic documentation or participants report	Assessment of accessibility	"Accessibility barriers" or "accessibility arrangements"
**Crosswalks**	Systematic documentation	According to pre-defined coding rules:<br>• Full accessibility : presence of both audible signal **and** tactile paving<br>• Partial accessibility: presence of both audible signal **or** tactile paving<br>• Inaccessibility: absence of both audible signal and tactile paving	• Accessibility barriers: inaccessible crosswalks<br>• Accessibility arrangements: full or partial accessibility
**Public transportation stations**	Systematic documentation	According to pre-defined coding rules:<br>• Accessibility: presence of audible signal<br>• Inaccessibility: absence of audible signal	• Accessibility barriers: inaccessible stations<br>• Accessibility arrangements: accessible stations
**Stairs**	Systematic documentation	According to pre-defined coding rules:<br>• Accessibility: presence of visual/tactile warning strips<br>• Inaccessibility: absence of visual/tactile warning strips	• Accessibility barriers: inaccessible stairs<br>• Accessibility arrangements: accessible stairs
**Narrow sidewalks**	Systematic documentation	According to pre-defined coding rules: a sidewalk not wide enough to allow two adults to walk comfortably next to each other	Accessibility barriers
**Unsmooth sidewalks**	Systematic documentation	According to pre-defined coding rules: Irregular, bumpy, uneven sidewalk	Accessibility barriers
**Proximity to construction sites**	Systematic documentation	According to pre-defined coding rules: proximity to construction sites with a lack of safety fence or safe walking spaces	Accessibility barriers
**Guardrails**	Systematic documentation	According to pre-defined coding rules: a barrier placed along the edge of a route at dangerous points	Accessibility arrangements
**Guidance marking**	Systematic documentation	According to pre-defined coding rules: tactile paving/paths on the pavement including truncated domes, detectable warnings, tactile tiles, tactile ground surface indicators, tactile walking surface indicators, or detectable warning surfaces	Accessibility arrangements
**Public fitness facilities**	Systematic documentation	According to pre-defined coding rules: safe fitness facilities in the public space (free access)	Accessibility arrangements
**Physical sidewalks obstacles**	Participants reports	Reported as problematic by participants in real-time (“obstacles-other” category): It included documentation of problematic locations of electric polls, lampposts, garbage cans, vegetation, mail boxes, street or store furniture as well as inexistent sidewalk, proximity to traffic, poor lighting or dangerous descents.	Accessibility barriers
